# Failure of productive infection of Mallards (*Anas platyrhynchos*) with H16 subtype of avian influenza viruses

**DOI:** 10.1111/irv.12275

**Published:** 2014-09-10

**Authors:** Sasan R Fereidouni, Timm C Harder, Anja Globig, Elke Starick

**Affiliations:** Friedrich-Loeffler-Institut (FLI)Greifswald-Insel Riems, Germany

**Keywords:** Avian influenza, H16 subtype, influenza pathogenesis, Mallard duck

## Abstract

**Background:**

Mallard ducks and other waterfowl represent the most important reservoirs of low pathogenic avian influenza viruses (LPAIV). In addition, mallards are the most abundant duck species in Eurasia that migrate over long distances. Despite extended wild bird monitoring studies over the past decade in many Eurasian countries and investigating hundreds of thousands of wild bird samples, no mallard duck was found to be positive for avian influenza virus of subtype H16 in faecal, cloacal or oropharyngeal samples. Just three cases of H16 infections in Anseriformes species were described worldwide. In contrast, H16 viruses have been repeatedly isolated from birds of the Laridae family.

**Objective:**

Here, we tested the hypothesis that mallards are less permissive to infection with H16 viruses.

**Methods:**

Groups of mallard ducks of different age were inoculated via the oculo-nasal-oral route with different infectious doses of an H16N3 AIV.

**Results:**

The ducks did not show any clinical symptoms, and no virus shedding was evident from cloacal and respiratory routes after experimental infection as shown by negative RT-qPCR results. In addition, all serum samples taken on days 8, 21 and 24 post-inoculation were negative by competitive NP-ELISA.

**Conclusions:**

This study provided evidence that mallards are resistant to infection with H16N3 LPAIV.

*Please cite this paper as:* Fereidouni *et al*. (2014) Failure of productive infection of Mallards (*Anas platyrhynchos*) with H16 subtype of avian influenza viruses. Influenza and Other Respiratory Viruses 8(6), 613–616.

## Introduction

Ducks, gulls and shorebirds constitute the primary natural reservoir of low pathogenic avian influenza viruses (LPAIV) which comprise, as far as avian hosts are concerned, 16 HA and 9 NA subtypes. Dabbling ducks are considered the main reservoir of LPAIV in nature. Mallards (*Anas platyrhynchos*) are the dabbling duck species from which LPAIVs have been isolated most frequently.^1^–^3^ In addition, mallards represent the most abundant duck species in Eurasia and migrate over long distances, for example, in Europe, along the East-Atlantic flyway. In experimental infection studies with different LPAIV strains, ducks were productively infected and produced and excreted large amounts of infectious virus progeny via faecal and respiratory routes.^4^–^7^ The AIV of subtype H16 was first reported in 2005 from black-headed gulls (*Larus ridibundus*) from Sweden.^8^ Since that time and despite of monitoring studies of several hundred thousands of wild bird samples, only a small number of H16 viruses have reportedly been isolated and characterized worldwide.^2^,^3^,^9^–^11^ Most of them were obtained from samples of members of the *Laridae* family. This might reflect a high susceptibility of members of this family to H16 viruses and/or a specific adaptation of this subtype to these hosts. The global populations of *Laridae* (gulls) and *Sternidae* families appear to be large and widespread enough to allow co-circulation of several influenza A virus lineages.^12^ Although other AIV subtypes are also occasionally detected in gulls, H13 and H16 viruses appear to circulate exclusively in *Laridae* species.^13^–^17^ H16 AIV infection in mallard ducks has only been reported twice (A/mallard/Gurjev/785/83/H16N3, A/mallard/Quebec/02916-1/2009/H16N3), despite the overall high susceptibility of mallards with most of the other fourteen HA subtypes of AIV.^18^–^20^ This might indicate an intrinsically low susceptibility of *Anseriformes*, especially mallards, to AIV of subtype H16. In addition, experimental infections of mallards with multiple H13 viruses, which are circulating in larid species, indicated that also influenza viruses of the H13 subtype are strongly host-adapted to gulls even if rare spillover into turkeys and mallards can occur.^4^

In this study, we tested the hypothesis that mallards are refractory to infection with AIV of subtype H16.

## Materials and methods

### Virus

The avian influenza virus strain A/herring gull/Germany/R3309/07 (H16N3) was used for the infection of mallards. The virus was isolated from a herring gull (*Larus argentatus*) during wild bird monitoring in Germany in 2007. A second passage of the virus in 10-day old SPF embryonated chicken eggs was produced for the animal experiments.

### Experimental design

Three groups of mallard ducks from a flock of captive-bred birds that was housed indoors at the quarantine stables of FLI were used in this study. The birds were handled and cared for in accordance with the Animal Protection guidelines, and legal approval of the trial had been granted (LALLF M-V/TSD/7221·3-2·5-003/11). All experiments were conducted under Biosafety Level 3 (BSL-3) conditions. Prior to inoculation, oropharyngeal and cloacal swabs were collected from each bird to ensure they were not acutely infected with any subtype of AIV at the start of the study. In addition, serum samples were collected to exclude a history of past AIV infection. In all experiments, each duck was inoculated via ocular, nasal and oropharyngeal routes with a total volume of one millilitre of virus suspension. The inoculum content was dispensed as following: one drop into the each eye and nostril and remaining suspension into the oropharyngeal area. A back titration of the infectivity titre of these suspensions was performed in MDCK cells. Health status of the birds was monitored twice daily, and swabs taken from the oropharynx and cloaca of the birds were immediately placed into the viral transport medium (MEM cell culture medium supplemented with 1 mL of Antibiotic-Antimycotic Solution, Sigma per 100 mL of medium). Swab samples were vortexed for 60 minutes at room temperature,^21^ before RNA was extracted using the Qiagen QIAmp Viral RNA Mini Kit. Eluted RNA was kept frozen at −70°C until further use.

#### Group 1

Twelve mallard ducks comprising two age groups of five 6-week-old and four 3-week-old ducklings were inoculated with 10^5^ TCID_50_ H16N3 virus. Three sentinel ducks (one 5-week-old and two 3-week-old) stayed in quarantine building and were mingled with the infected ducks two days after inoculation. Oropharyngeal and cloacal swabs were collected daily starting 48 hours after inoculation. The experiment was terminated at 8 days post-inoculation (dpi) when blood samples were collected for serological testing, and internal organs were evaluated for gross pathological lesions.

#### Group 2

Ten adult (3-year-old) mallard ducks were selected. Four of these ducks had previously been injected intramuscularly for production of antisera with AIV antigen of H3N8, H5N3 or H16N3 subtypes at an age of 10 weeks and had produced detectable specific antibodies for 4 months. These birds were selected to test that any previous exposure to influenza viruses, priming for homo- or heterosubtypic immunity, might trigger the immune system to produce antibodies against H16N3 virus. Eight birds were inoculated with 10^5^ TCID_50_ of the H16N3 virus. Two sentinel ducks were mingled with the infected animals from 2 dpi onwards. Oropharyngeal and cloacal swabs were collected on 2, 3, 4, 6 and 8 dpi. The experiment was terminated at 24 dpi when blood samples were taken, and post-mortem evaluation of internal organs was carried out.

#### Group 3

Nine 6-weeks-old mallard ducks hatched and raised at the FLI quarantine facilities were selected. Eggs were purchased from a commercial hatchery. At the BSL-3 facilities, five ducks were inoculated with 10^5^ TCID_50_ and four with 10^6^ TCID_50_ of the H16N3 virus. Monitoring and sampling was as in group 2, and the experiment was terminated at 21 dpi. The experiment was carried out to confirm previous results using higher doses of the virus and to monitor inoculated mallards for a longer period.

### Real-time reverse transcription-PCR (RT-qPCR)

RNA of cloacal and oro-pharyngeal swab samples was examined by RT-qPCR using primers targeting the influenza A virus nucleoprotein (NP) gene.^22^ Reactions were prepared with the AgPath-ID One-Step RT-PCR Kit (AgPath, Applied Biosystems, Darmstadt, Germany) and run on a MX3005P Real-Time PCR platform (Stratagene, Germany). In all tests, negative RNA preparation controls and negative and positive RT-qPCR controls were included. In addition, an internal control (IC-2) was included from the step of RNA preparation onwards.^23^ RT-qPCR was considered positive in swab samples with a cycle threshold value (C_q_) lower than 36.^24^

### Competitive ELISA

Serum samples were tested in a competitive ELISA targeting influenza A virus NP antibodies following the manufacturer's instructions (ID Screen®, Influenza A NP Antibody Competition, ID.VET, Montpellier, France). The results were expressed as percentage inhibition (PI) calculated according to the formula:

PI value = [1-(OD sample/mean OD negative)] × 100

Based on the PI value, the samples were classified as negative if the PI value was above 50% and positive if the PI value was below 45%.

### Haemagglutination inhibition (HI) assay

Detection of haemagglutination inhibiting (HI) antibodies was based on standard protocols^25^ using four haemagglutination units of antigen prepared from AIV strain A/herring gull/Germany/R3309/07(H16N3).

### Haemagglutination (HA) assay (receptor-binding assay)

The HA activity of gull- and duck-derived AIV isolates was compared using red blood cell (RBC) samples collected from chickens and mallard ducks based on standard protocols^25^ using antigen prepared from strains A/herring gull/Germany/R3309/07(H16N3), A/herring gull/Germany/R2792/06 (H16N3), A/mallard/Germany/R1647/07(H4N6), A/herring gull/Germany/R2788/2006 (H16N3), A/black-headed gull/Sweden/5/99 (H16N3), A/wild bird/wv1136-40/03 (H13N6), A/black-headed gull/Germany/R2622/06 (H13N2), A/ black-headed gull Germany/R2064/06 (H13N8), A/mallard/Germany/R1740/07 (H4N6) and A/mute swan/Germany/R2927/07 (H6N8).

## Results and discussion

### Animal status before LPAIV exposure

All ducks were virus-negative prior to inoculation as revealed negative results by RT-qPCR. In addition, ducks were serologically negative to influenza A antigens tested by ELISA indicating that the birds were not recently exposed to AIV. Four ducks that had been used for serum production against LPAIV isolates three years earlier also tested NP-seronegative.

### Mallards were refractory to experimental infection with AIV H16N3

Back titration of viral inocula confirmed that the intended doses of infectious virus had indeed been applicated, that is 10^5^ TCID_50_ H16N3 virus for the experiments 1–3 and 10^6^ TCID_50_ for second part of the last experiment. All birds of the three groups remained clinically healthy up to the end of the respective observation periods after inoculation. The total of 456 cloacal and oro-pharyngeal swab samples taken from all birds of all groups revealed negative results in RT-qPCR indicating that no detectable virus shedding after virus inoculation occurred in any of the mallard ducks. Post-mortem pathological investigations showed no gross pathological findings. Tissue samples taken from animals of group 1 were not evaluated for presence of virus antigen by immunohistochemistry (IHC), because it has been already shown that virus antigen is only detectable by IHC, when birds are shedding high titres of virus,^19^ which was clearly not the case in this study. In addition, all naive sentinel mallards remained virus-negative in this study which indicates a lack of transmission and emphasize the fact that virus shedding did not occur in ducks inoculated with H16N3 virus. Daoust *et al*.^20^ also showed lack of significant virus shedding after experimental infection of mallard ducks with a gull-adopted H13N6 LPAIV.

None of the sera obtained at the end of the observation periods of 8, 21 and 24 dpi, respectively, reacted seropositive in either the competitive NP-specific ELISA or in HI tests using homologous virus antigen. We have previously shown that high titres of specific antibodies were detectable by ELISA and HI one week after inoculation of mallard ducks with LP H5N2 AIV.^26^

The replication potential of H16 viruses in ducks has been studied here for the first time. However, neither clinical nor virological or serological evidence for a successful experimental infection of mallard ducks of different age was obtained. These results, in addition to the findings of global wild bird surveillance studies, suggest that mallards are resistant to infection with AIV of subtype H16. The current study has been carried out using a single H16N3 isolate. Thus, it cannot be excluded that other H16 isolates may be able to infect and replicate in mallards. There are two entries in GenBank that claim detection of H16N3 in mallards (A/mallard/Gurjev/785/83, A/mallard/Quebec/02916-1/2009) so that this possibility cannot be rendered fully unlikely.

### HA tests using chicken and duck RBC

Yamnikova *et al*.^27^ described fundamental differences in the receptor-binding site (RBS) between HA proteins of subtype H13 originating from *Laridae* and other AIV subtypes from *Anatidae*. Nine amino acid exchanges in the HA of H13 viruses from gulls compared with duck-origin viruses of different subtypes result in a transformation of the receptor phenotype of H13 viruses isolated from gulls. Fouchier *et al*.^8^ demonstrated that seven of these nine different amino acids were also present in the RBS of the H16 HA protein. The different structure of the virus RBS could thus provide a molecular explanation for the putative host restriction of H16 and H13 viruses.

We tried to assess this putative effect by performing HA assays with gull-adapted H13 and H16 AIVs in comparison with duck-specific AIV using chicken and duck RBCs. The HA titres of all investigated viruses were the same or differed only by one log level when using chicken versus duck RBCs. Unfortunately, no Laridae erythrocytes were available for further comparisons which compromise the conclusions. In addition, *in vitro* binding studies of the viruses to Laridae and Anseriformes tissue should be carried out to rule out the possibility that RBCs cannot reflect receptor-binding specificities of these viruses.

The crystal structure of the H16 haemagglutinin precursor HA_0_ as resolved by Lu *et al*.^28^ revealed a unique alpha-helix structure in the endoproteolytic cleavage site which is believed to reduce the accessibility for trypsin-like proteases that are essentially required for endoproteolytical processing of the HA of other AIV subtypes replicating efficiently in *Anseriformes*.

In addition to virus attachment mediated by the HA protein, host species-specific adaptation markers may be present also in other viral proteins: Tonnessen *et al*.^29^ investigated the amino acid composition of the six ‘internal’ gene segments of H16 and H13 viruses and found *Laridae*-specific signatures especially in the nucleoprotein (NP) and in the non-structural protein 1 (NS1). They concluded that the host specificity of H13 and H16 AIV may not solely be determined by HA-mediated receptor recognition and interaction but that internal viral proteins such as NS1 and NP may have an additional influence.

In summary, here we have shown, by infection experiments, that mallard ducks were resistant for infection with AIV of subtype H16.
